# Genetic Analysis of Flooding Tolerance in an Andean Diversity Panel of Dry Bean (*Phaseolus vulgaris* L.)

**DOI:** 10.3389/fpls.2018.00767

**Published:** 2018-06-06

**Authors:** Ali Soltani, Samira MafiMoghaddam, Atena Oladzad-Abbasabadi, Katelynn Walter, Patrick J. Kearns, Jose Vasquez-Guzman, Sujan Mamidi, Rian Lee, Ashley L. Shade, Janette L. Jacobs, Martin I. Chilivers, David B. Lowry, Phillip McClean, Juan M. Osorno

**Affiliations:** ^1^Department of Plant Sciences, North Dakota State University, Fargo, ND, United States; ^2^Plant Resilience Institute, Michigan State University, East Lansing, MI, United States; ^3^Department of Microbiology and Molecular Genetics, Michigan State University, East Lansing, MI, United States; ^4^Genome Sequencing Center, HudsonAlpha Institute for Biotechnology, Huntsville, AL, United States; ^5^Department of Plant, Soil and Microbial Sciences, Michigan State University, East Lansing, MI, United States; ^6^Department of Plant Biology, Michigan State University, East Lansing, MI, United States

**Keywords:** common bean, flooding, abiotic stress, anoxia, waterlogging, GWAS

## Abstract

Climate change models predict temporal and spatial shifts in precipitation resulting in more frequent incidents of flooding, particularly in regions with poor soil drainage. In these flooding conditions, crop losses are inevitable due to exposure of plants to hypoxia and the spread of root rot diseases. Improving the tolerance of bean cultivars to flooding is crucial to minimize crop losses. In this experiment, we evaluated the phenotypic responses of 277 genotypes from the Andean Diversity Panel to flooding at germination and seedling stages. A randomized complete block design, with a split plot arrangement, was employed for phenotyping germination rate, total weight, shoot weight, root weight, hypocotyl length, SPAD index, adventitious root rate, and survival score. A subset of genotypes (*n* = 20) were further evaluated under field conditions to assess correlations between field and greenhouse data and to identify the most tolerant genotypes. A genome-wide association study (GWAS) was performed using ~203 K SNP markers to understand the genetic architecture of flooding tolerance in this panel. Survival scores between field and greenhouse data were significantly correlated (*r* = 0.55, *P* = 0.01). Subsequently, a subset of the most tolerant and susceptible genotypes were evaluated under pathogenic *Pythium* spp. pressure. This experiment revealed a potential link between flooding tolerance and *Pythium* spp. resistance. Several tolerant genotypes were identified that could be used as donor parents in breeding pipelines, especially ADP-429 and ADP-604. Based on the population structure analysis, a subpopulation consisting of 20 genotypes from the Middle American gene pool was detected that also possessed the highest root weight, hypocotyl length, and adventitious root development under flooding conditions. Genomic regions associated with flooding tolerance were identified including a region on Pv08/3.2 Mb, which is associated with germination rate and resides in vicinity of *SnRK1.1*, a central gene involved in response of plants to hypoxia. Furthermore, a QTL at Pv07/4.7 Mb was detected that controls survival score of seedlings under flooding conditions. The association of these QTL with the survivability traits including germination rate and survival score, indicates that these loci can be used in marker-assisted selection breeding to improve flooding tolerance in the Andean germplasm.

## Introduction

Flooding adversely affects plants by reducing ATP synthesis that is accompanied by carbohydrate starvation. Carbohydrate limitation results from suboptimal photosynthesis and carbohydrate metabolism. Elevated levels of reactive oxygen species (ROS, Blokhina et al., 2003) as well as disruption in root hydraulic conductance and mineral absorption (Holbrook and Zwieniecki, 2003; Tournaire-Roux et al., 2003) have been reported for plants under flooding conditions. Furthermore, excess water facilitates disease spread, especially fungal and oomycete pathogens that affect roots. Several studies reported a high correlation of flooding stress with soybean (*Glycine max* L.) stem/root rot damage (caused by *Phytophthora sojae*; Cornelious et al., 2005; Helms et al., 2007; Nguyen et al., 2012; Valliyodan et al., 2016), or with *Pythium* spp. in dry bean (*Phaseolus vulgaris* L.; Li et al., 2016).

Plants have evolved different mechanisms to cope with flooding stress. To deal with hypoxic conditions that result from flooding, plants use either escape or quiescence strategies (Colmer and Voesenek, 2009). The escape strategy is characterized by plants developing new organs or modifying the morphology of existing organs to facilitate oxygen intake from the surrounding environment. The escape strategy includes development of adventitious roots, shoot elongation, and aerenchyma formation (Colmer and Voesenek, 2009). When utilizing the quiescence strategy, plants actively suppress any morphological changes related to the escape strategy to minimize energy consumption. During quiescence, carbohydrate catabolism provides the energy for critical pathways required for plant survival. These strategies vary among species and are often defined by the dominant flooding regime in the environment to which a particular species has adapted. Due to intrinsic fitness trade-off between the escape and quiescence strategies, both cannot be adopted at the same time. Colmer and Voesenek (2009) have argued that the escape strategy is more important in environments with prolonged flooding, while the quiescence strategy is more important in environments that experience transient flooding. Similarly, a wide diversity of responses to flooding stress have been reported in crops (Setter and Laureles, 1996; Valliyodan et al., 2016; Soltani et al., 2017). Although some crops, such as lowland rice (*Oryza sative* L.), are well-adapted to flooding, the majority of economically important crops, including common bean, are susceptible.

Common bean is the most important edible legume, providing protein, fiber, macro-, and micro-nutrients for direct human consumption worldwide (Messina, 1999). Morphological, biochemical, and molecular evidence suggests that *P. vulgaris* originated in Mexico, then a subset of this species migrated to Central America and the Andes in South America (Bitocchi et al., 2012). Humans are responsible for multiple independent domestication events of common bean, in both Middle America and South America (Gepts et al., 1986). Eco-geographic variation within the Middle American and Andean gene pools resulted in the creation of races (Singh et al., 1991). At least three races were characterized in the Middle American gene pool, including Durango, Jalisco, and Mesoamerica (Singh et al., 1991). Within the Andean gene pool, three major races include Nueva Granada, Chile, and Peru. Modern market classes of beans are derived from each gene pool and are defined primarily based on the seed color, shape, and size (Singh et al., 2007). The commonly produced market classes of beans in the U.S., pinto, great northern, pink, small red, black, and navy, are from the Middle American gene pool. Kidney and cranberry beans are the most common market classes from the Andean gene pool commercially grown in the U.S. (Singh et al., 2007).

While common bean production is adversely affected by a variety of biotic and abiotic stresses, excess water has recently been reported as the main production issue in North Dakota, the largest producer of dry bean in the U.S. (Knodel et al., 2015, 2016, 2017). During the growing season of 2016, 16,700 acres (28% of the total dry bean farmlands in North Dakota) were negatively impacted by excess water (Knodel et al., 2017). Excess water is often accompanied by diseases such as white mold (*Sclerotinia sclerotiorum*), common bacterial blight (caused by *Xanthomonas axonopodis* pv. *phaseoli*), and root rot (caused by *Pythium* spp.; Knodel et al., 2016). The presences and virulence of parasitic *Pythium* (Oomycota) species are dependent on the edaphic and environmental factors (Rojas et al., 2017; Rossman et al., 2017). In North Dakota, dry bean production occurs mostly in the Red River Valley. The majority of the soils in this region drain poorly with the water table at or near the soil surface (Miller and White, 1998). These soils are usually characterized by a high content of expansive clay (USDA-NRCS., 2017). Heavy rainfall incidents at the early growth stages of common bean (germination, emergence, and establishment) causes flooding in these poorly-drained farmlands, which result in lower plant densities and significant seed yield losses. Tile drainage is often used as a solution but it is expensive (Kandel et al., 2013). Therefore, the use of genetic tolerance may be a more efficient and less expensive option to cope with flooding stress assuming there is genetic variation for tolerance within the bean germplasm.

To develop an efficient breeding strategy for dry bean, understanding the variation of flooding tolerance among genotypes and its underlying genetic architecture is crucial. Genomic loci associated with flooding tolerance have been identified in several legume species, including soybean (VanToai et al., 2001; Sayama et al., 2009; Osman et al., 2013). A recent study on flooding tolerance in a panel of Middle American germplasm revealed that responses of genotypes to flooding at early stages (germination and seedling) could be grouped based on eco-geographical races (Soltani et al., 2017). Further, several genomic regions associated with flooding tolerance were detected, including a region on Pv08 that was associated with several flooding tolerance traits at the seedling stage.

Here, we evaluate the responses of Andean common bean germplasm to flooding. By conducting the study on the Andean germplasm, we could compare and contrast our findings with results from the distinct Middle American germplasm (Soltani et al., 2017). The current study was designed to address two major objectives: (1) to identify the most tolerant Andean genotypes under the excess water condition to be used in genetic improvement, and (2) to identify the genomic regions associated with the tolerance response within Andean germplasm under flooding conditions. The results of this study pave the way for the development of new dry bean varieties that can better tolerate flooded environments.

## Materials and methods

### Plant material

A subset of photoperiod insensitive genotypes (*n* = 277) from the Andean Diversity Panel (ADP; Cichy et al., 2015) were evaluated in this study. These genotypes were collected from several geographical regions, including Africa (*n* = 147), North America (*n* = 83), Central America (*n* = 26), South America (*n* = 12), and Eurasia (*n* = 9). The purple-podded cultivar Royalty was included in this experiment as a tolerant check based on previous results (Soltani et al., 2017).

### Phenotyping procedure in the greenhouse

A randomized complete block design (RCBD) with a split plot arrangement was used to screen the panel under greenhouse conditions at the germination and seedling developmental stages. This design included two levels of treatment (flooded vs. non-flooded), where genotypes were considered as sub-plots. Three and four replications were considered for evaluation at germination and seedling stages, respectively.

Phenotyping at both stages was performed at the NDSU greenhouse complex in Fargo, ND following the flooding screening method described in Soltani et al. (2017). To evaluate the panel at the germination stage, ten seeds were sown in a sandy soil. Designated flooding treatment trays were completely submerged in the water for 1 day while control trays were watered regularly. Excess water was drained and germination rate was evaluated for both flooded and non-flooded plots after 2 weeks. For evaluation at seedling stage, two seedlings per genotype were grown under well-drained conditions until reaching the V2 stage. Stress designated pots were transferred to plastic flats that were filled with water up to 2 cm above the soil level. Plants were exposed to this flooding stress for 10 days, at which point the water was drained. Chlorophyll content was measured using the SPAD 502 Chlorophyll Meter (Spectrum Technologies, Inc.), and adventitious root formation was visually scored from 0 (no adventitious root) to 5 (highest adventitious root). The same visual scoring scale (0–5) was used for the survival score in which 0 indicates dead plants and 5 indicate healthy plant with no obvious visual stress symptoms. Following scoring, the seedlings were harvested, washed, and dried in oven for 2 weeks at 38°C. Additional traits were quantified on dried plants, including total weight (TW, g), shoot weight (SW, g), root weight (RW, g), and hypocotyl length (HL, cm).

### Phenotyping procedure in the field

To evaluate how tolerant and susceptible germplasm performs under field conditions, a group of selected genotypes (based on the greenhouse screening) were grown in the field at NDSU in the spring/summer of 2016. Ten susceptible (ADP-4, ADP-19, ADP-10, ADP-62, ADP-670, ADP-648, ADP-428, ADP-462, ADP-440, ADP-5) and 10 tolerant genotypes (ADP-630, ADP-626, ADP-651, ADP-618, ADP-603, ADP-650, ADP-672, ADP-673, ADP-604, Royalty) were planted in a randomized complete block design (RCBD) in a split-plot arrangement, with three replicates per genotype. Each replicate consisted of a two-row plot, with 80 seeds per row. The field was located on the NDSU campus in Fargo, ND (46° 53′58″ N, 96°48′52.3″ W). The soil in this field contains 2% sand, 45% silt, and 53% clay. All the plots were grown under normal well-drained conditions until V2 developmental stage, at which point the designated plots were continuously flooded using a sprinkler irrigation system. After 10 days of flooding, a survival score of each plot was visually estimated from 0 (all plants were dead) to 5 (all plants survived). On the same day, SPAD index of three random plants per line was measured using the SPAD 502 Chlorophyll Meter. Above-ground tissues of three random plants per plot were collected, dried for 2 weeks and weighed.

### *Pythium* isolation and pathogenicity test

Based on the greenhouse screening results, two genotypes susceptible to excess water conditions (ADP-4 and ADP-41) were grown in a greenhouse at Michigan State University. Plants were grown in top soils obtained from Michigan (MI) and North Dakota (ND). Two distinct soil sources were used in this experiment to account for the potential *Pythium* species variation between regions. Following flooding for 1 week at V2 stage, roots and stems were surface sterilized in fresh 10% bleach and rinsed clean of bleach with sterile water. Plant material was placed on 20% clarified V8 agar amended with ampicillin (250 mg L^−1^), rifampicin (10 mg L^−1^), pimaricin (5 mg L^−1^), and terraclor (133.3 mg L^−1^, Jeffers and Martin, 1986). Plates were incubated for 3 days at room temperature and colonies with *Pythium*-like growth were subcultured on 20% V8 agar until a pure culture was obtained. DNA from each pure *Pythium*-like isolate (total *n* = 18, *n* = 6 from North Dakota, *n* = 12 from Michigan) was extracted by repeated freeze-thaw and the internal transcribed spacer (ITS) region was amplified with the primer pair ITS1F and ITS4R (White et al., 1990) following the conditions published previously (Kageyama et al., 1997) using high-fidelity Pfu Turbo DNA Polymerase (Agilent, Santa Clara, SA, USA). PCR amplification was checked with gel electrophoresis and amplicons were purified with the Wizard SV Gel and PCR Clean-up System (Promega, Madison, WI, USA). Amplicons were quantified fluorometrically with the Qubit (Thermo Fisher, Waltham, MA, USA) and sequenced at the Michigan State Genomics Core. ITS sequences were deposited in NCBI under accession numbers: from MH109027 to MH109043. ITS sequences were identified with BLASTn against the nt database. The hits with the lowest E-value were selected as the most significant match.

To evaluate the interaction of flooding and *Pythium* on plant performance, we conducted a controlled growth chamber experiment. Five tolerant (ADP-673, ADP-604, ADP-672, ADP-650, ADP-603) and five susceptible (ADP-4, ADP-41, ADP-122, ADP-20, ADP-19) genotypes were selected based on SPAD values measured during the initial greenhouse experiment. Seed surfaces were sterilized using 2% bleach and seeds were sown in fine vermiculite under controlled growth chamber condition with 25/20 °C day/night temperatures and a 16-h day photoperiod. At the V2 growth stage, plants were exposed to three treatments: flooding without any inoculation, flooding in presence of *P. ultimum*, and flooding in presence of *P. irregulare. P. ultimum* and *P. irregulare* were the isolates recovered from ND and MI soils, respectively (see Results). Inoculum of both species was prepared by growing isolates on a semi-selective medium, CMA-PARP (Jeffers and Martin, 1986) amended with benomyl (10 μg/mL, CMA-PARPB) for 2 or 3-days. Sterile white millet grain (1.6 Kg) was inoculated with 1 cm^2^ plugs from two 100 × 15 mm culture plates. Inoculated white millet was incubated at room temperature for 14 days, with mixing every other day to allow even colonization of all grains. The inoculum was air-dried prior to use and three grams of inoculum was added to each pot. Four replicates were evaluated for each treatment/genotype combination. After 1 week, chlorophyll content (SPAD) of primary leaves was measured using a MultiSpeQ (Kuhlgert et al., 2016).

### Statistical analysis

Due to non-homogenous error variances, the germination rate, survival scores, and adventitious root formation data were transformed using the Box-Cox method (Box and Cox, 1964). All the greenhouse and field phenotypic data were analyzed by fitting mixed model ANOVAs in SAS 9.3 using PROC MIXED. In this model, genotypes and treatments were considered as fixed effects and replicates as random effects.

The flooding index was calculated for each trait/genotype using Equation (1):

(1) $$\begin{array}{l} Flooding\;index\;\left(FI\right)=\frac{Flooding\;value\;-\;non\;flooding\;value}{non\;flooding\;value} \end{array}$$

To select the most tolerant lines from the whole panel, the following selection index was developed:

(2) $$\begin{array}{l} \text{Selection index}=\left(2\times \text{survival score}\right)+|\text{SPAD FI}|+|\text{Root FI}|\\ +\text{adventitious root rate} \end{array}$$

In this index, a 2-fold higher weight was assigned to the survival score, as it plays a more important role in breeding for flooding tolerance. The Pearson correlation coefficients were calculated using psych (Revelle, 2016) and plotted using corrplot package (Wei and Simko, 2016) in R (R Development Core Team., 2011). Biplot was plotted by using ggbiplot in R environment. Broad-sense heritability and genetic variances were estimated by the method described by Holland et al. (2003).

### Population structure and phylogenetic relationships

For structure and phylogenetic analysis, a set of SNP markers with linkage disequilibrium (LD) *r*^2^ of 0.10 or lower was selected using SNPhylo pipeline (Lee et al., 2014). SNP markers were generated by using a low-pass sequencing protocol described by Schröder et al. (2016). The libraries sequenced at Hudson Alpha Institute for Biotechnology. Fastx barcode splitter and Fastx trimmer were used for splitting the barcodes and trimming the data respectively. A default quality threshold of 20 and a minimum trimmed length of 80 bp were applied using sickle (Joshi and Fass, 2011). GATK UnifiedGenotyper (McKenna et al., 2010; v3.3) with minimum confidence threshold of 30, was applied to call the SNPs. Population structure was analyzed using the STRUCTURE V2.3.4 (Pritchard et al., 2000). This analysis was performed using 10,000 burn-in and 100,000 Markov chain Monte Carlo repetitions. To define the number of subpopulations (K) in the panel, K was tested from 1 to 10 with five iterations for each run. The optimal K was then identified using the nonparametric Wilcoxon test between adjacent runs (McClean et al., 2012). The estimated cluster coefficient matrices from different replications were then imported to CLUMPP (Jakobsson and Rosenberg, 2007) to obtain a single permutated matrix for each K. The visual representation of populations was plotted using the STRUCTURE PLOT (Ramasamy et al., 2014). The phylogenetic tree was constructed using SNPhylo and Maximum Likelihood algorithm.

### Genome-wide association study

GAPIT (Lipka et al., 2012) was used for GWAS. A total of 203,574 SNP markers with MAF ≥ 5% were used in this analysis. Multiple models were tested for each trait as explained previously in Moghaddam et al. (2016) under both flooded and non-flooded conditions. The first two principal components were used to account for population structure (cumulatively controlled ~25% of the variation). The best model was selected based on the lowest mean square difference (MSD, Mamidi et al., 2011). The final Manhattan plots were created using the R package “gap” (Zhao, 2007). Significant SNPs in GWAS were determined using two cutoff thresholds based on the empirical distribution of the *P*-values after 100 bootstraps: (i) a stringent cutoff where SNPs fall in the lower 0.01 percentile tail and (ii) a relaxed cutoff where SNPs fall in the 0.1 percentile tail. The phenotypic variation explained by most significant markers (which was defined as the markers with lowest *P*-value) in the best model was calculated based on the likelihood-ratio-based *r*^2^ (Sun et al., 2010) using the genABLE package in R (Aulchenko et al., 2007), which controls for structure and/or relatedness. A search for candidate genes was performed using the annotation data from the V 2.1 of the bean genome sequence (phytozome.org) within ± 100 Kb from the most significant marker.

## Results

### Distribution of data and analysis of variance

**Figure 1:**
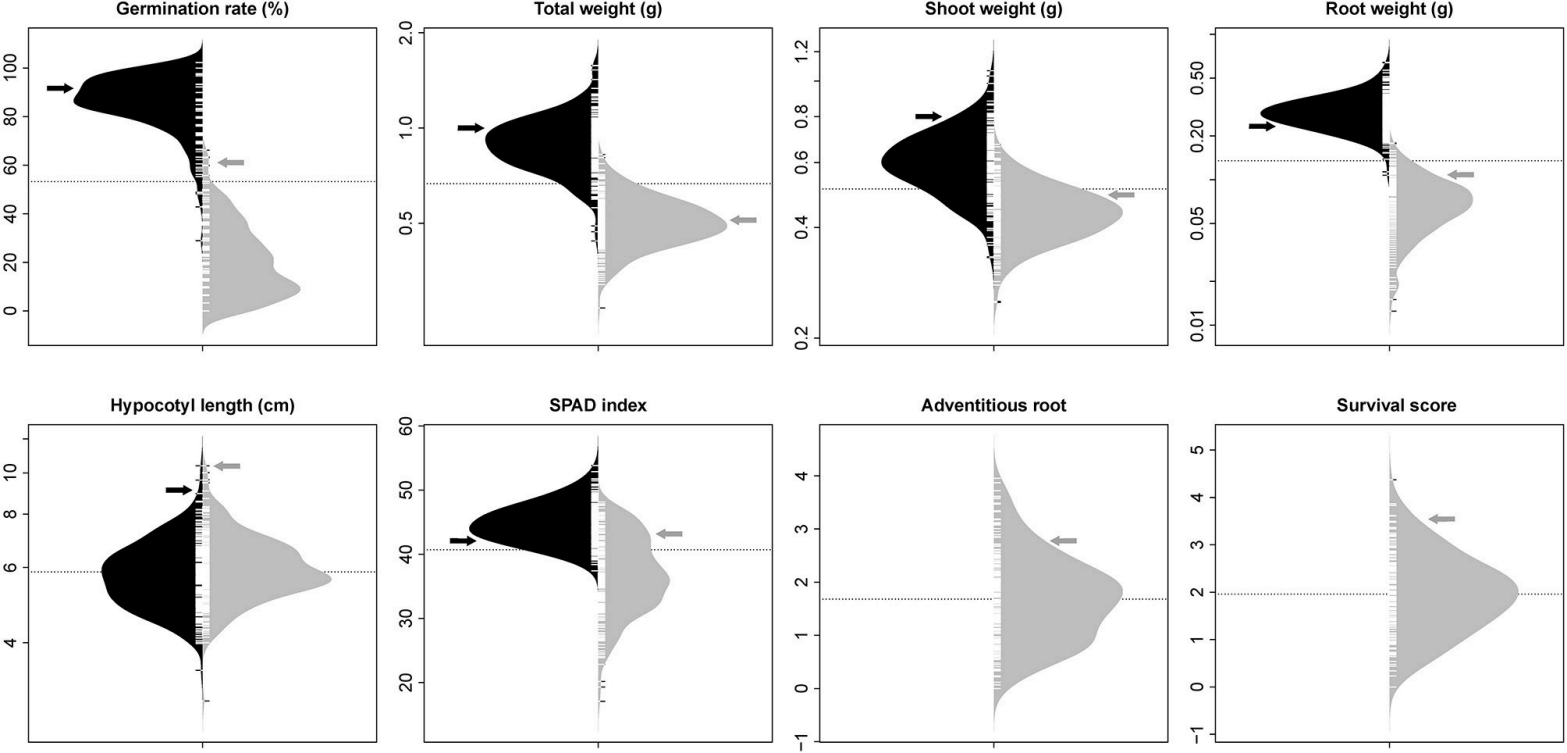
Bean plots represent the distribution of traits in non-flooded (black) and flooded (gray) conditions. The units of y-axis for each trait was mentioned after the name of the trait on the top of each graph. The arrows indicate the values of tolerant check (Royalty) for each condition. The dotted lines indicates the total means under both flooding and control conditions. No adventitious root or survival reduction was observed in control condition.

The phenotypic distribution of each trait was plotted separately for each treatment (Figure 1). Significant differences between treatments were detected for four traits including germination rate, total weight, shoot weight, and root weight (Table 1). However, SPAD index and hypocotyl length were not affected by the treatment. Significant differences were detected among genotypes for all the measured traits regardless of the treatment. Significant interactions between treatment × genotype were found for germination rate, total weight, root weight, and SPAD index.

**Table 1:** Results of ANOVA for eight traits.

	**Treatment**					**Genotype**			**Treatment × Genotype**
	**Mean square**	**Mean** ± **se**		**CV%**		**Mean square**	**Mean ± se**	**CV%**	**Mean square**
**Trait**		**Non-flooded**	**Flooded**	**Non-flooded**	**Flooded**				
Germination rate	8271.93^**^	85.813 ± 0.692	20.617 ± 0.896	13	72	3.57^***^	53.215 ± 1.497	66	1.78^***^
Root weight	27.30^*^	0.294 ± 0.005	0.072 ± 0.002	28	45	0.02^***^	0.183 ± 0.005	70	0.01^**^
Shoot weight	15.89^*^	0.608 ± 0.007	0.438 ± 0.004	20	17	0.06^***^	0.523 ± 0.005	25	0.02
Total weight	84.84^*^	0.903 ± 0.011	0.510 ± 0.005	20	18	0.12^***^	0.707 ± 0.010	34	0.04^**^
SPAD	35918	44.681 ± 0.184	36.585 ± 0.369	7	17	109.43^***^	40.633 ± 0.267	16	78.18^*^
Hypocotyl length	26.38	5.862 ± 0.064	6.082 ± 0.067	18	18	8.65^***^	5.972 ± 0.046	18	0.75
Adventitious root^†^	–	−	1.684 ± 0.049	–	49	0.32^***^	1.684 ± 0.049	49	–
Survival score	–	−	1.959 ± 0.049	–	42	0.72^***^	1.959 ± 0.049	42	–

* *Significant at 0.05 probability level*.

** *Significant at 0.01 probability level*.

*** *Significant at 0.001 probability level. Adventitious root was observed only in flooded condition*.

Several tolerant genotypes were identified that can be used as parental lines in breeding programs (Tables S1, S2). The most tolerant genotype at germination is ADP-429 (PR9920-171, Table S1), which has a Cranberry seed type. ADP-604 (1062-V98, Table S2) which is a light red kidney bean showed significant levels of tolerance under both flooding and *Pythium* presence at the seedling stage. This line can be used in breeding programs as a donor parent.

### Population structure

**Figure 2:**
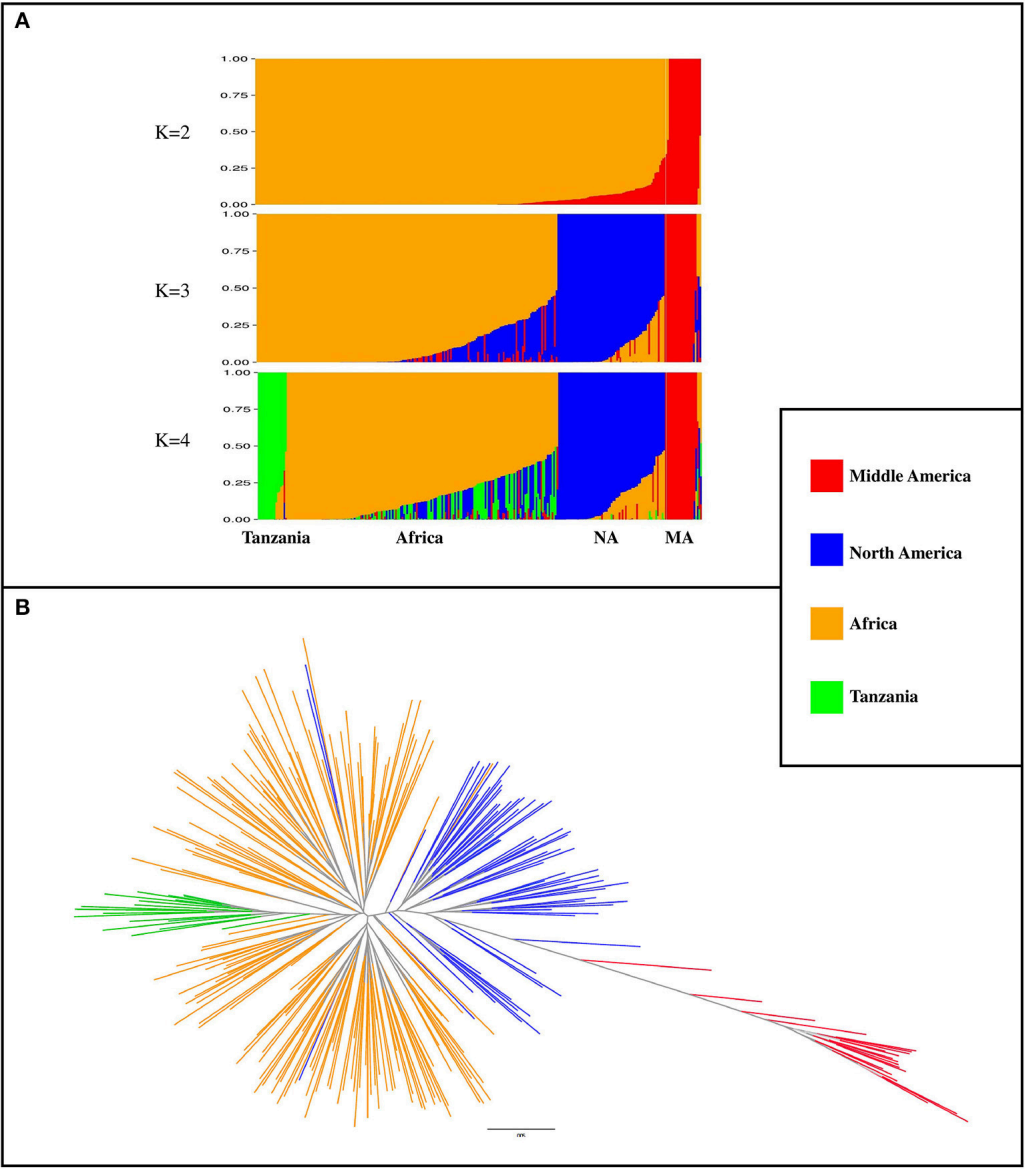
Population classification using a subset of 3,668 markers that had LD *r*^2^ ≤ 0.1 (markers in linkage equilibrium). **(A)** Population structure from *K* = 2 to *K* = 4. K indicates the number of subpopulations. NA, North America; MA, Middle America **(B)** phylogenetic tree of genotypes, constructed by using SNPhylo. The colors of subpopulations were the same as population structure plot (*K* = 4).

A total of 3,668 SNP markers with local pairwise LD *r*^2^ ≤ 0.1 were identified. This subset of markers was used for both structure analysis and phylogenetic tree construction. Genotypes with subpopulation membership (*Q*) estimates lower than 0.80 were considered as “admixed”. At *K* = 2, a subpopulation of 20 genotypes with a Middle American origin was identified with an average subpopulation membership value of 0.99 (Figure 2A). At *K* = 3, the Northern American germplasm was separated from the African and Middle American genotypes (Figure 2A). Based on the Wilcoxin test, *K* = 4 was identified as the optimum number of subpopulations (Figure 2A). At *K* = 4, four subpopulations (Table S3) were identified, including (1) Tanzania: a subset of 18 genotypes that all originated from Tanzania; (2) Africa: the majority of genotypes (*n* = 173; 71%) originated from Africa; (3) North America: a subset of 67 genotypes that contains 89% North American genotypes; and (4) Middle America: a subset of 20 genotypes with Middle American origin. A phylogenetic tree was constructed to evaluate the relationship among genotypes and subpopulations (Figure 2B). Based on this analysis, genotypes with Middle American origin were phylogenetically closer to genotypes with North American origin.

### Correlation between population structure and phenotypes

To investigate the potential relationship of population structure and the traits considered here, admixed genotypes (genotypes with *Q* estimates lower than 0.80) were removed from each subpopulation and the means of each subpopulation were estimated. In the non-flooded condition, the North American subpopulation had significantly higher values for germination rate, total weight, shoot weight, and root weight (Table 2). For hypocotyl length, the North American and Middle American subpopulations were at least one centimeter longer compared with the other two subpopulations. The Tanzania subpopulation, with 45.82 units, had the highest value for SPAD index compared with other subpopulations in non-flooded condition. In flooded condition, Middle American subpopulation had the highest hypocotyl length (7.21 cm), and adventitious root formation (1.96). However, North American subpopulation had the highest values for total weight (0.58 g), shoot weight (0.50 g), SPAD index (38.21), and survival score (2.17) in the flooding treatment.

**Table 2:** Trait means for the four major subpopulations.

	**Non-flooded**				**Flooded**			
	**Tanzania**	**Africa**	**North America**	**Middle America**	**Tanzania**	**Africa**	**North America**	**Middle America**
Germination rate (%)	84.09a^†^	84.36a	90.92b	85.26a	19.47a	20.87a	20.43a	23.08a
Total weight (g)	0.87a	0.88a	1.04b	0.83a	0.50a	0.45b	0.58c	0.48ab
Shoot weight (g)	0.58a	0.59ab	0.70c	0.54b	0.44a	0.40b	0.50c	0.39b
Root weight (g)	0.28a	0.28a	0.33b	0.29a	0.06a	0.05a	0.08b	0.08b
Hypocotyl length (cm)	5.26a	5.21a	6.79b	6.84b	5.49a	5.45a	6.78b	7.21c
SPAD index	45.82a	43.24b	44.65c	42.56b	36.61a	34.18b	38.21a	33.31b
Adventitious root ‡	–	–	–	–	1.59a	1.56a	1.74b	1.96c
Survival score	–	–	–	–	1.68a	1.80ab	2.17b	1.86ab

† *Means followed by the same letter in each row are not significantly different at 0.05 probability level. ‡ Adventitious root was observed only in flooded condition*.

**Figure 3:**
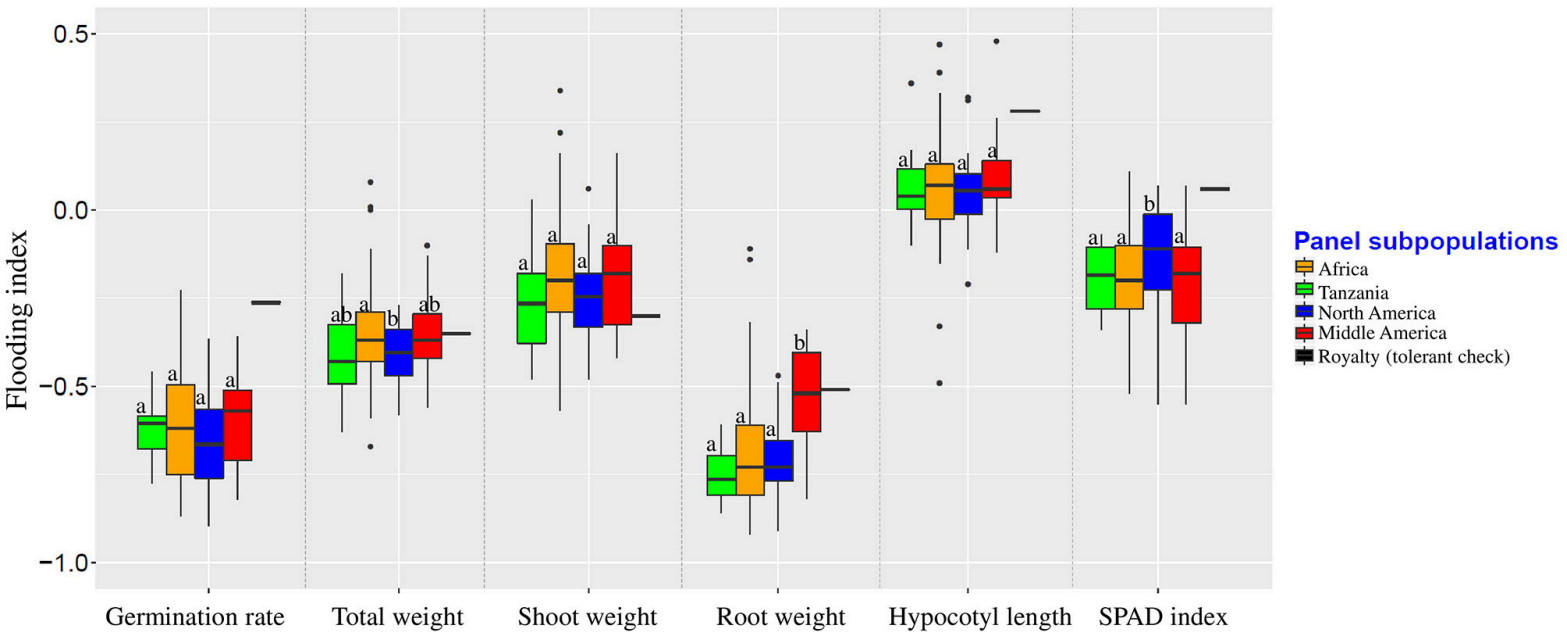
Flooding indices of different subpopulations. Groups were color-coded based on the results of population structure plot (*K* = 4, Figure 2). Boxplots indicated by the same letter for each trait are not significantly different at 0.05 probability level.

Population structure was not correlated with the flooding indices of germination rate, shoot weight, and hypocotyl length (Figure 3). Flooding index for chlorophyll content (SPAD index) was significantly higher in North American subpopulation, However, a significant higher flooding index for root weight was detected in Middle American subpopulation.

### Broad-sense heritability estimates and genetic variances

Broad-sense heritability and genetic variance of each trait were estimated in both flooded and non-flooded conditions (Table 3). Results indicate that the heritability estimate of germination rate in flooded condition was two times higher than the non-flooded condition (67.55 and 29.85, respectively). However, the heritability of SPAD index in non-flooded condition (65.69) was three times higher than the flooded condition (22.70).

**Table 3:** Broad-sense heritabilities and genetic variances of traits.

**Trait**	**Parameter**	**Non-flooded**	**Flooded**	**Flooding index**
Germination rate	H^2^^†^	29.85 ± 7.41	67.55 ± 3.38	45.98 ± 5.71
	$\sigma_{G}^{2}$	0.1681	0.8344	0.0103
Total weight	H^2^	53.91 ± 4.58	54.84 ± 4.43	0.00 ± 0.00
	$\sigma_{G}^{2}$	0.0175	0.0046	0.0000
Shoot weight	H^2^	51.03 ± 4.87	56.01 ± 4.32	0.00 ± 0.00
	$\sigma_{G}^{2}$	0.0074	0.0030	0.0000
Root weight	H^2^	36.21 ± 6.35	43.17 ± 5.56	31.35 ± 6.86
	$\sigma_{G}^{2}$	0.0024	0.0004	0.0090
Hypocotyl length	H^2^	80.22 ± 1.95	81.04 ± 1.87	0.00 ± 0.00
	$\sigma_{G}^{2}$	0.9330	1.0062	0.0000
SPAD index	H^2^	65.69 ± 3.38	22.70 ± 7.59	21.09 ± 7.85
	$\sigma_{G}^{2}$	6.2106	8.6574	0.0040
Adventitious root ‡	H^2^	–	44.16 ± 4.76	–
	$\sigma_{G}^{2}$	–	0.0420	–
Survival score	H^2^	–	22.00 ± 6.22	–
	$\sigma_{G}^{2}$	–	0.0498	–

† *Broad-sense heritability was quantified using $\frac{\sigma_{G^{2}}}{\sigma_{G}^{2}+\frac{\sigma_{e}^{2}}{r}}$, in which $\sigma_{G}^{2}$ is the genetic variance, $\sigma_{e}^{2}$ is the residual variance, and r is the number of replicates. ‡ Adventitious root was observed only in flooded condition*.

Genetic variances of total weight, shoot weight, and root weight were higher (4, 2.5 and 6-fold, respectively) in non-flooded condition. However, genetic variance was higher in flooded condition for germination rate and SPAD index (5 and 1.5-fold, respectively). The genetic variances of hypocotyl length were similar between flooded and non-flooded conditions.

### Correlation and biplot analysis

**Figure 4:**
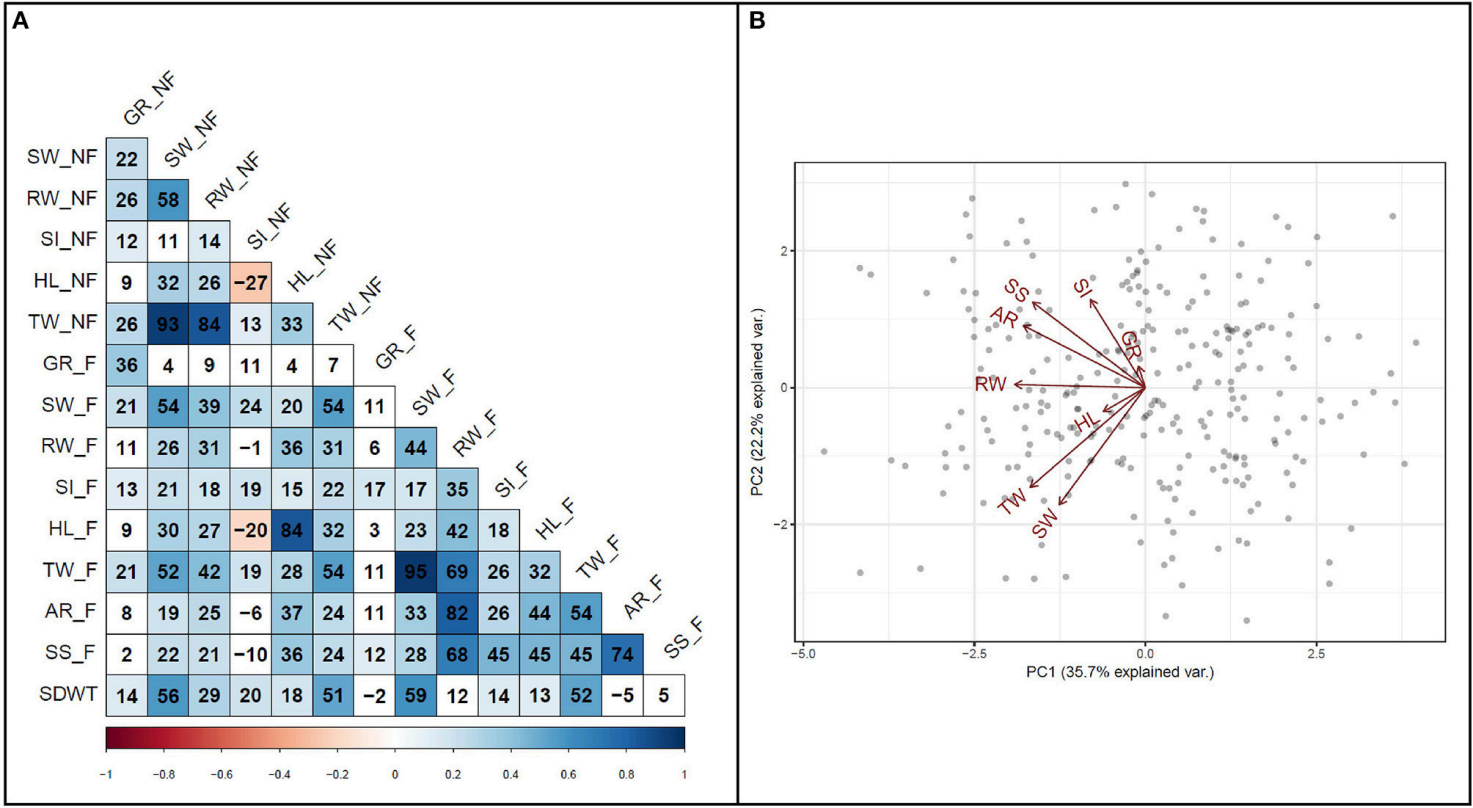
Relationship among traits. **(A)** Pearson correlation coefficients among traits measured under flooded and non-flooded. Correlation coefficients multiplied by 100. **(B)** Biplot representing the relationship among different flooding indices used to separate genotypes. Traits evaluated in non-flooded and flooded conditions represented by _N and _F, respectively after the name of the trait. TW, total weight; SW, shoot weight; RW, root weight; HL, hypocotyl length; SI, SPAD index; GR, germination rate; AR, adventitious root formation; SS, survival score and SDWT, seed weight.

To investigate the relationship among variables, a correlation analysis was performed among all traits (Figure 4A). A strong positive correlation (*r* = 0.74, *P* < 0.001) was detected between survival score and degree of adventitious root formation in flooded condition. Furthermore, a strong positive correlation (*r* = 0.82, *P* < 0.001) was detected between adventitious root formation and root weight. Biplot analysis on flooding indices also revealed the existence of strong positive correlations among adventitious root formation, survival score, and SPAD index (Figure 4B). The results also indicated that hypocotyl length and germination rate were among the variables with the lowest variance in the PCA.

### Field evaluation and *pythium* pathogenicity test

A subset of Andean genotypes were grown under the flooded and non-flooded conditions in the field and evaluated for three traits including survival score, SPAD index, and shoot weight (Table S4). Among the traits evaluated under the flooding condition, shoot weight (SW_GH), and SPAD index (SI_field) had the highest correlation (*r* = 0.65, *P* = 0.002) between greenhouse and field data (Figure S1). A significant but moderately strong positive correlation (*r* = 0.55, *P* = 0.01) was detected between survival scores measured in the field and in the greenhouse. In contrast, no significant correlation was detected between SPAD index evaluated in the greenhouse and the field.

The lack of a significant correlation between greenhouse and field SPAD data suggested that an unmeasured variable might be affecting the flooding response. To investigate the potential role of pathogen infestation under flooding conditions, susceptible genotypes were grown under flooded conditions in two different soil sources (MI and ND). In total, 18 *Pythium*-like isolates were obtained. Twelve isolates from MI soil were similar (96 or 98% identity) to *P. irregulare* based on their ITS sequences (Table S5). *Pythium*-like isolates obtained from the ND soil were identified as being closely related to either *P. ultimum* (95% identity, 5 isolates) or *P. sylvaticum* (97% identity, one isolate).

Ten bean genotypes were subjected to a pathogenicity test for the two dominant *Pythium* isolates (*P. irregulare* and *P. ultimum*). The results indicate that tolerant genotypes have on average 16% significantly higher SPAD values across treatments (Figure S2A). In particular, the differences between tolerant and susceptible genotypes for SPAD index are more drastic in the presence of *P. irregulare* (Figure S2B).

### Genome-wide association study

**Figure 5:**
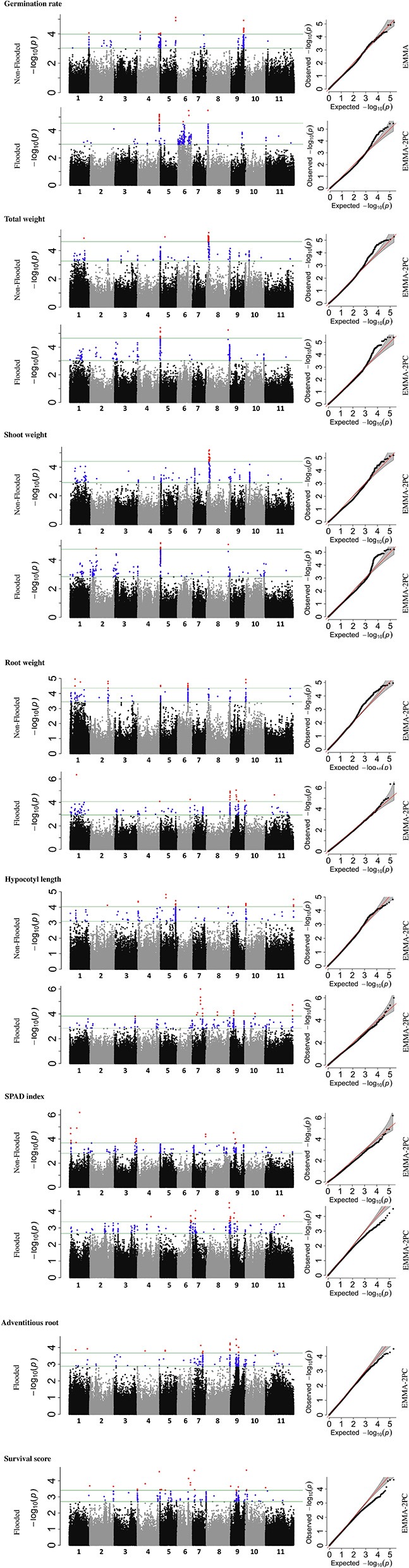
Manhattan and corresponding Q-Q plots represents the genetic architecture of different traits under flooded and non-flooded condition. Position of loci associated with seven traits in non-flooded and flooded conditions are represented. Blue dots in Manhattan plots represent SNPs falling between 0.01 and 0.1 percentile tail of the empirical distribution of *P*-values. Red dots represent SNPs that pass the 0.01 percentile. The name of the model selected for each trait/ treatment was mentioned on the right side of Q-Q plots.

To identify the genetic architecture of traits under flooded condition, GWAS was performed for all traits in both flooded and non-flooded conditions (Figure 5 and Table 4). Overall, 45 significant peaks were identified for eight traits across treatments, from which 19 and 22 peaks were identified only under non-flooded or flooded condition, respectively (Table 4). Four peaks were identified in both treatment conditions. The peak on Pv07/24.4, which was associated with hypocotyl length, possessed the highest *r*^2^ (*r*^2^ = 17.22) among flooding responsive peaks. The peak on Pv02/32.0 that was associated with root weight had the highest *r*^2^ (*r*^2^ = 18.02) among non-flooded peaks (Table 4). A region on Pv08/59–62.3 Mb was detected that was associated with five traits under flooded condition, including total weight, shoot weight, SPAD index, adventitious root formation, and the survival score. Potential candidate genes for these loci are reported in Table S6.

**Table 4:** Major loci associated with traits in non-flooded and flooded conditions.

	**Trait**	**Locus abbr**.	**Chr**	**Position**	**Class^†^**	**-log10 (*P*-value)**	***r^2^***
**GERMINATION RATE**							
	Non-flooded	Pv01/49.8	1	49,760,345	NF	4.07	13.33
		Pv04/6.4	4	6,446,043	NF	4.12	12.40
		Pv05/36.5	5	36,523,875	NF	5.12	15.68
		Pv09/33.4	9	33,436,855	NF	4.92	16.33
							36.26
	Flooded	Pv04/45.7	4	45,724,128	F	5.19	13.03
		Pv06/16.0	6	16,034,362	F	5.47	14.31
		Pv08/3.2	8	3,260,637	F	5.48	14.30
							20.59
**TOTAL WEIGHT**							
	Non-flooded	Pv08/7.5	8	7,494,340	NF	5.28	16.51
	Flooded	Pv05/1.2	5	1,163,339	both ‡	5.41	15.89
		Pv08/59.1	8	59,069,546	F	5.25	15.35
							22.17
**SHOOT WEIGHT**							
	Non-flooded	Pv08/7.5	8	7,494,340	NF	5.20	15.77
	Flooded	Pv05/1.2	5	1,163,367	both	5.21	15.89
		Pv08/59.1	8	59,069,546	F	5.11	15.58
							21.34
**ROOT WEIGHT**							
	Non-flooded	Pv01/28.5	1	28,497,505	NF	4.77	17.11
		Pv02/32.0	2	32,055,514	NF	4.80	18.02
		Pv05/1.3	5	1,296,877	NF	4.53	16.86
		Pv06/14.9	6	14,869,116	NF	4.66	17.46
		Pv10/1.0	10	1,023,101	NF	4.93	17.41
							36.97
	Flooded	Pv08/62.3	8	62,282,561	F	4.95	12.90
		Pv09/13.5	9	13,549,503	F	5.04	15.26
							21.64
**HYPOCOTYL LENGTH**							
	Non-flooded	Pv04/0.2	4	177,101	NF	4.37	12.09
		Pv05/13.9	5	13,923,936	NF	4.82	12.63
		Pv05/35.7	5	35,759,769	NF	4.42	11.54
		Pv10/0.4	10	384,405	NF	4.23	11.02
		Pv11/52.9	11	52,955,904	both	4.50	11.77
							39.75
	Flooded	Pv03/46.9	3	46,914,727	F	3.82	10.31
		Pv07/24.4	7	24,450,920	F	6.00	17.22
		Pv09/7.8	9	7,809,795	F	4.25	11.62
		Pv11/52.9	11	52,955,972	both	4.72	13.29
							33.05
**SPAD INDEX**							
	Non-flooded	Pv01/3.9	1	3,931,202	NF	4.89	13.74
		Pv03/50.0	3	50,059,460	NF	4.03	10.73
		Pv07/38.3	7	38,274,271	NF	4.39	12.44
		Pv09/11.5	9	11,514,939	NF	4.51	9.86
							29.94
	Flooded	Pv06/24.3	6	24,294,817	F	3.73	12.21
		Pv07/8.1	7	8,083,989	F	4.04	10.04
		Pv08/61.1	8	61,080,306	F	4.51	11.41
							27.74
**ADVENTITIOUS ROOTS §**							
	Flooded	Pv07/28.7	7	28,690,630	F	3.78	9.70
		Pv08/62.3	8	62,270,633	F	4.26	10.24
		Pv09/13.5	9	13,549,503	F	4.49	11.47
		Pv09/20.2	9	20,230,630	F	4.01	10.30
							33.60
**SURVIVAL SCORE**							
	Flooded	Pv02/47.3	2	47,348,500	F	3.65	10.73
		Pv04/46.8	4	46,852,214	F	4.56	12.15
		Pv07/4.7	7	4,735,092	F	4.65	13.73
		Pv09/19.1	9	19,108,550	F	3.62	6.02
		Pv10/1.5	10	1,471,633	F	4.66	13.06
							37.82

* *Classification was based on detection of QTL in each treatment. NF = QTL only detected in the non-flooded condition. F = QTL detected in flooding condition and both = QTL were detected in both treatments (non-flooded vs. flooded). ‡ The QTL on Pv05/1.2 associated with total and shoot weight was identified in both flooded and non-flooded conditions. Under the non-flooded condition, the significant markers are falling between 0.01 and 0.1 percentile tail of the empirical distribution of P-values, however in flooded condition, it passes the 0.01 percentile. For more clarifications, refer to Figure 5. § Adventitious root was observed only in flooded condition*.

## Discussion

Flooding tolerance traits in both Andean and Middle American gene pools are consistently complex, being controlled by several loci. In the Andean gene pool (current study), higher genetic diversity was detected under flooding condition for traits that are related to plant fitness, including germination rate and leaf chlorosis that was measured by SPAD. Leaf chlorosis can be considered as a trait related to fitness since chlorotic plants will produce less offspring. However, morphometric traits, including total weight, shoot weight, and root weight, had higher genetic variability ($\sigma_{G}^{2}$) in non-flooded condition. These results are similar to the results of a flooding experiment conducted with the Middle American diversity panel (Soltani et al., 2017). In general, it has been proposed that genetic diversity of traits related to fitness should be higher in stress conditions, with the opposite expected for morphometric traits (Visscher et al., 2008).

### Survivability of andean germplasm under excess water condition at germination stage

At the germination stage, the Andean diversity panel performed poorly under flooding condition. Indeed, just 1% of the genotypes had a germination rate equal or greater than the tolerant check (Royalty).

A genetic factor for germination rate under flooded conditions was located at Pv08/3.2 Mb, ~1.5 Kbp upstream of *Phvul.008G039400*, homologous to *SnRK1.1*. Plant SnRK family members are classified into three major groups (SnRK1, SnRK2, and SnRK3) and play a crucial role in responses to different abiotic stresses (Arzani and Ashraf, 2016). SnRK1 genes, particularly SnRK1.1 and SnRK1.2, were identified as the central components of regulatory mechanisms under hypoxic condition (Baena-González et al., 2007; Loreti et al., 2017). In rice, it was shown that SnRK1A is involved in triggering the starvation inducible α-amylase gene activity, which consequently leads to better germination under flooded conditions (Lee et al., 2009). snRK1A and trehalose-6-phosphate function synergistically and enable efficient germination and plant establishment under hypoxic conditions (Loreti et al., 2016). Interestingly, a region at Pv02/41 Mb in the Middle American diversity panel was associated with germination rate under flooding (Soltani et al., 2017). This region was located near trehalose-6-phosphate synthase. Thus, while the main genomic regions that control seed germination under flooding conditions are different between the Andean and Middle American gene pools, the functional genes might be involved in the same biological pathway.

### Response of andean genotypes to flooding stress at seedling stage

Traits associated with survivability have proved useful in the identification of the most tolerant genotypes to different abiotic stresses, such as salinity (Rezaei et al., 2017). Therefore, a weighted selection index was developed in which a 2-folds higher weight was assigned to the survival score. Selection indices greater than or equal to ten were chosen as the most tolerant lines. These lines comprise 6% of the whole panel and include Royalty, the tolerant check.

Furthermore, the significant correlation between greenhouse and field data for the survival score indicates that greenhouse evaluations can be a useful selection tool in breeding programs. The non-significant correlation between field and greenhouse for SPAD index may reflect the influence of other factors, such as presence of different *Pythium* species or variations in soil nutrient content in different geographical regions.

Several genes, involved in leaf senescence and chlorophyll degradation were detected in vicinity of a QTL associated with survival score under flooding condition. The gene *ICS2*, involved in salicylic acid biosynthesis, was detected at Pv10/1.5 Mb. This gene is expressed constitutively in vascular tissues and contributes to defense mechanism of plants against diseases (Macaulay et al., 2017). Indeed, several experiments on soybean reported the co-localization of the flooding-related QTL with disease related genes, particularly for *P. sojae* (Cornelious et al., 2005; Helms et al., 2007; Nguyen et al., 2012; Valliyodan et al., 2016). In dry bean, flooding triggers pathogenesis by *Pythium* spp. (Li et al., 2016). In our previous study on flooding responses in Middle American germplasm (Soltani et al., 2017), symptoms of root diseases such as damping-off, root rot, and root necrosis were not detected. In contrast, these symptoms were observed in this study for the majority of flooding susceptible Andean genotypes. *Pythium* spp. were isolated from susceptible genotypes grown under flooding condition, which indicates the potential role of root diseases triggered by excess soil water. This finding suggests that breeding for flooding tolerance in the Andean germplasm should include enhancing resistance to root diseases.

Our results also indicate that under flooding conditions, susceptibility of genotypes to *Pythium* infestation, is associated with a significant decrease in chlorophyll content. In general, it is known that Andean genotypes are more susceptible to root rot diseases, particularly *Fusarium* (Bilgi et al., 2008). The response of Andean genotypes to different *Pythium* spp. should be investigated in future studies.

Adventitious root formation is highly correlated with survival score and potentially contributed to survivability of plants under flooding condition. Adventitious root formation in flooding conditions is an escape strategy by which plants improve the oxygen delivery from surrounding environments to the internal tissues as well as improving nutrition absorption (Steffens and Rasmussen, 2016). Formation of adventitious roots is promoted by higher concentration of auxin and lower levels of cytokinin (Steffens and Rasmussen, 2016). Several genes, involved in auxin and cytokinin metabolism and translocation were identified in close proximity of detected QTL for adventitious root and root weight. For instance, a locus associated with root weight and adventitious root formation was identified on Pv09/13.5 Mb. This locus is in close proximity of *SLOMO*, which is involved in spatial distribution of auxin in plants (Lohmann et al., 2010). *CKX7*, which is involved in cytokinin catabolism (Köllmer et al., 2014) was also located in this region. Another significant QTL was detected at Pv08/62.3 Mb is associated with root weight and adventitious root formation under flooding condition. This QTL resides at ~43 Kb downstream of Root Growth Factor 1 Insensitive 4 (RGI4, Ou et al., 2016). This gene belongs to leucine-rich receptor-like protein kinase (LRR-RLKs) and function as a receptor for Root Growth Factor 1 (*RGF1*). *RGF1* is a secreted peptide hormone that regulates root meristem development in *Arabidopsis*. Mutants of *RGI4* display a drastic reduction in root formation in *Arabidopsis* (Ou et al., 2016).

### Comparison of response to flooding in andean and middle american germplasm

Based on the population structure analysis, *K* = 4 was defined as the optimal number of subpopulations. The Middle American subpopulation was the most distant subpopulation from the other three. The members of this group, which were collected from Africa and the Caribbean, were reported to originate from the Middle American gene pool (Cichy et al., 2015). Better performance of this group, particularly for root weight, may indicate the existence of root rot resistance allele(s), which originated from Middle American gene pool. Middle American genotypes were phylogenetically closer to genotypes collected mainly from North America. The Middle American subgroup possessed the longest hypocotyl length and highest adventitious root formation. A longer internode length has previously been observed in bean varieties adapted to lowland conditions, where the occurrence of excess water is more frequent (Aidar et al., 2000). This morphology might be an adaptive strategy for keeping the leaves and pods away from soil moisture and potential diseases. Interestingly, Royalty, which is one of the most tolerant genotypes to flooding possessed a long hypocotyl compared with other genotypes. Furthermore, flooding indices of root weight for Middle American genotypes were significantly higher than other subpopulations, which might be related to their ability to change their root architecture and formation of more adventitious roots.

## Conclusion

Survivability of Andean genotypes is greatly compromised by the presence of excess moisture in the soil. Lower survivability of Andean beans can be the direct result of root rot pathogens, particularly *Pythium* spp. that can spread efficiently in moist soils. Several QTL were identified that can be used for marker-assisted selection. These QTL can be considered as Andean-specific QTL since they were not detected in our previous study with the majority of Middle American germplasm. The QTL region at Pv08/59–62.3 Mb, which is associated with several traits under flooding conditions, could prove useful in marker-assisted selection. Furthermore, QTL at Pv07/4.7 Mb can be used to improve the survival score of the plants under flooding condition. Biparental QTL analysis using the distinct parental genotypes can be highly valuable in revealing rare alleles that are not detectable in GWAS. Furthermore, bulked-segregant analysis (BSA, Michelmore et al., 1991) can be employed to identify significant QTL. This approach was recently used to develop molecular markers linked to *Bean common mosaic virus* (BCMV) resistance in common bean (Bello et al., 2014). Screening variation in wild beans, as well as different closely related species, is indispensable for detecting novel sources of tolerance to flooding that can be introgressed into the existing Andean germplasm. Comparative expression and metabolomics analysis between the most tolerant and susceptible genotypes will be crucial to reveal the mechanistic pathways controlling responses to flooding.

## Author contributions

AS, KW, and JV performed greenhouse and field evaluations. PM, RL, and SM contributed to genotyping of the panel. AS, SMM, and AO contributed to GWAS. PK and ALS contributed to *Pythium* isolation and ITS sequencing. JJ and MC contributed to *Pythium* pathogenecity test. AS, JO, and DL designed the experiment and prepared the manuscript. All authors reviewed and edited the manuscript.

### Conflict of interest statement

The authors declare that the research was conducted in the absence of any commercial or financial relationships that could be construed as a potential conflict of interest.

## References

[B1] Aidar H. Thung M. Kluthcouski J. de Oliveira I. Cabrera J. (2000). Bean Production in the Lowland Tropic with Sub-Irrigation. Annual Report of the Bean Improvement Cooperative, East Lansing, MI, 134–135.

[B2] Arzani A. Ashraf M. (2016). Smart engineering of genetic resources for enhanced salinity tolerance in crop plants. CRC Crit. Rev. Plant Sci. 35, 146–189. 10.1080/07352689.2016.1245056

[B3] Aulchenko Y. S. Ripke S. Isaacs A. van Duijn C. M. (2007). GenABEL: an R library for Genome-Wide association analysis. Bioinformatics 23, 1294–1296. 10.1093/bioinformatics/btm108 17384015

[B4] Baena-González E. Rolland F. Thevelein J. M. Sheen J. (2007). A central integrator of transcription networks in plant stress and energy signalling. Nature 448, 938–942. 10.1038/nature06069 17671505

[B5] Bello M. H. Moghaddam S. M. Massoudi M. McClean P. E. Cregan P. B. Miklas P. N. (2014). Application of *in silico* bulked segregant analysis for rapid development of markers linked to Bean common mosaic virus resistance in common bean. BMC Genomics 15:903. 10.1186/1471-2164-15-903 25326146 PMC4210609

[B6] Bilgi V. N. Bradley C. A. Khot S. D. Grafton K. F. Rasmussen J. B. (2008). Response of dry bean genotypes to Fusarium root rot, Caused by *Fusarium solani* f. sp. phaseoli, under field and controlled conditions. Plant Dis. 92, 1197–1200. 10.1094/PDIS-92-8-1197 30769492

[B7] Bitocchi E. Nanni L. Bellucci E. Rossi M. Giardini A. Zeuli P. S.. (2012). Mesoamerican origin of the common bean (*Phaseolus vulgaris* L.) is revealed by sequence data. Proc. Natl. Acad. Sci. U.S.A. 109, E788–E796. 10.1073/pnas.1108973109 22393017 PMC3325731

[B8] Blanvillain R. Kim J. H. Wu S. Lima A. Ow D. W. (2009). OXIDATIVE STRESS 3 is a chromatin-associated factor involved in tolerance to heavy metals and oxidative stress. Plant J. 57, 654–665. 10.1111/j.1365-313X.2008.03717.x 18980652

[B9] Blokhina O. Virolainen E. Fagerstedt K. V. (2003). Antioxidants, oxidative damage and oxygen deprivation stress: a review. Ann. Bot. 91, 179–94. 10.1093/aob/mcf118 12509339 PMC4244988

[B10] Box G. E. P. Cox D. R. (1964). An analysis of transformations. J. R. Stat. Soc. Ser. B 26, 211–252.

[B11] Chang L. Ramireddy E. Schmülling T. (2013). Lateral root formation and growth of *Arabidopsis* is redundantly regulated by cytokinin metabolism and signalling genes. J. Exp. Bot. 64, 5021–5032. 10.1093/jxb/ert291 24023250 PMC3830484

[B12] Cichy K. A. Porch T. G. Beaver J. S. Cregan P. Fourie D. Glahn R. P. (2015). A *Phaseolus vulgaris* diversity panel for andean bean improvement. Crop Sci. 55, 2149–2160. 10.2135/cropsci2014.09.0653

[B13] Colmer T. D. Voesenek L. A. C. J. (2009). Flooding tolerance: suites of plant traits in variable environments. Funct. Plant Biol. 36, 665–681. 10.1071/FP09144 32688679

[B14] Cornelious B. Chen P. Chen Y. de Leon N. Shannon J. G. Wang D. (2005). Identification of QTLs underlying water-logging tolerance in soybean. Mol. Breed. 16, 103–112. 10.1007/s11032-005-5911-2

[B15] Frelet-Barrand A. Kolukisaoglu H. U. Plaza S. Rüffer M. Azevedo L. Hörtensteiner S.. (2008). Comparative mutant analysis of *Arabidopsis* ABCC-Type ABC transporters: AtMRP2 contributes to detoxification, vacuolar organic anion transport and chlorophyll degradation. Plant Cell Physiol. 49, 557–569. 10.1093/pcp/pcn034 18325934

[B16] Garapati G.-P. Xue P. Munné-Bosch S. Balazadeh S. (2015). Transcription factor ATAF1 in *Arabidopsi*s promotes senescence by direct regulation of key chloroplast maintenance and senescence transcriptional cascades. Plant Physiol. 168, 1122–1139. 10.1104/pp.15.00567 25953103 PMC4741325

[B17] Ge Y. Yan F. Zourelidou M. Wang M. Ljung K. Fastner A.. (2017). SHADE AVOIDANCE 4 is required for proper auxin distribution in the hypocotyl. Plant Physiol. 173, 788–800. 10.1104/pp.16.01491 27872246 PMC5210748

[B18] Gepts P. Osborn T. C. Rashka K. Bliss F. A. (1986). Phaseolin-protein variability in wild forms and landraces of the common bean (*Phaseolus vulgaris*): evidence for multiple centers of domestication. Econ. Bot. 40, 451–468. 10.1007/BF02859659

[B19] Hála M. Soukupová H. Synek L. Žárský V. (2010). *Arabidopsis* RAB geranylgeranyl transferase β-subunit mutant is constitutively photomorphogenic, and has shoot growth and gravitropic defects. Plant J. 62, 615–627. 10.1111/j.1365-313X.2010.04172.x 20180921

[B20] Helms T. C. Werk B. J. Nelson B. D. Deckard E. (2007). Soybean tolerance to water-saturated soil and role of resistance to *Phytophthora sojae*. Crop Sci. 47, 2295–2302. 10.2135/cropsci2007.03.0175

[B21] Holbrook N. M. Zwieniecki M. A. (2003). Plant biology: water gate. Nature 425:361. 10.1038/425361a 14508474

[B22] Holland J. B. Nyquist W. E. Cervantes-Martinez C. T. (2003). Estimating and interpreting heritability for plant breeding, in Plant Breeding Reviews, ed Janick J. (John Wiley & Sons, Inc.), 9–112.

[B23] Horie Y. Ito H. Kusaba M. Tanaka R. Tanaka A. (2009). Participation of chlorophyll b reductase in the initial step of the degradation of light-harvesting chlorophyll a/b-protein complexes in Arabidopsis. J. Biol. Chem. 284, 17449–17456. 10.1074/jbc.M109.008912 19403948 PMC2719385

[B24] Jakobsson M. Rosenberg N. A. (2007). CLUMPP: a cluster matching and permutation program for dealing with label switching and multimodality in analysis of population structure. Bioinformatics 23, 1801–1806. 10.1093/bioinformatics/btm233 17485429

[B25] Jeffers S. N. Martin S. B. (1986). Comparison of two media selective for *Phytophthora* and *Pythium* species. Plant Dis. 70, 1038–1043. 10.1094/PD-70-1038

[B26] Joshi N. Fass J. (2011). Sickle: A Sliding-Window, Adaptive, Quality-Based Trimming Tool for Fastq Files (Version 1.33). Available online at: https://github.com/najoshi/sickle.

[B27] Kageyama K. Ohyama A. Hyakumachi M. (1997). Detection of *Pythium ultimum* using polymerase chain reaction with species-specific primers. Plant Dis. 81, 1155–1160. 10.1094/PDIS.1997.81.10.1155 30861711

[B28] Kandel H. J. Brodshaug J. A. Steele D. D. Ransom J. K. Desutter T. M. Sands G. R. (2013). Subsurface drainage effects on soil penetration resistance and water table depth on a clay soil in the Red River of the North Valley, USA. Agric. Eng. Int. 15, 1–10.

[B29] Knodel J. J. Beauzay P. B. Endres G. J. Franzen D. W. Kandel H. J. Markell S. G. (2017). 2015 Dry Bean Grower Survey of Production, Pest Problems and Pesticide Use in Minnesota and North Dakota. North Dakota State University.

[B30] Knodel J. J. Beauzay P. B. Franzen D. W. Kandel H. J. Markell S. G. Osorno J. M. (2015). 2014 Dry Bean Grower Survey of Production, Pest Problems and Pesticide Use in Minnesota and North Dakota. Fargo, ND: North Dakota State University.

[B31] Knodel J. J. Beauzay P. B. Franzen D. W. Kandel H. J. Markell S. G. Osorno J. M. (2016). 2015 Dry Bean Grower Survey of Production, Pest Problems and Pesticide Use in Minnesota and North Dakota. North Dakota State University

[B32] Köllmer I. Novák O. Strnad M. Schmülling T. Werner T. (2014). Overexpression of the cytosolic cytokinin oxidase/dehydrogenase (CKX7) from Arabidopsis causes specific changes in root growth and xylem differentiation. Plant J. 78, 359–371. 10.1111/tpj.12477 24528491

[B33] Kuhlgert S. Austic G. Zegarac R. Osei-Bonsu I. Hoh D. Chilvers M. I.. (2016). MultispeQ Beta: a tool for large-scale plant phenotyping connected to the open PhotosynQ network. R. Soc. Open Sci. 3:160592. 10.1098/rsos.160592 27853580 PMC5099005

[B34] Lee K. W. Chen P. W. Lu C. A. Chen S. Ho T. H. Yu S. M. (2009). Coordinated responses to oxygen and sugar deficiency allow rice seedlings to tolerate flooding. Sci. Signal. 2:ra61. 10.1126/scisignal.2000333 19809091

[B35] Lee T. H. Guo H. Wang X. Kim C. Paterson A. H. (2014). SNPhylo: a pipeline to construct a phylogenetic tree from huge SNP data. BMC Genomics 15:162. 10.1186/1471-2164-15-162 24571581 PMC3945939

[B36] Levesque-Tremblay G. Havaux M. Ouellet F. (2009). The chloroplastic lipocalin AtCHL prevents lipid peroxidation and protects *Arabidopsis* against oxidative stress. Plant J. 60, 691–702. 10.1111/j.1365-313X.2009.03991.x 19674405

[B37] Li Y. P. You M. P. Norton S. Barbetti M. J. (2016). Resistance to Pythium irregulare root and hypocotyl disease in diverse common bean (*Phaseolus vulgaris*) varieties from 37 countries and relationships to waterlogging tolerance and other plant and seed traits. Eur. J. Plant Pathol. 146, 147–176. 10.1007/s10658-016-0901-2

[B38] Lipka A. E. Tian F. Wang Q. Peiffer J. Li M. Bradbury P. J.. (2012). GAPIT: genome association and prediction integrated tool. Bioinformatics 28, 2397–2399. 10.1093/bioinformatics/bts444 22796960

[B39] Lohmann D. Stacey N. Breuninger H. Jikumaru Y. Muller D. Sicard A.. (2010). SLOW MOTION is required for within-plant auxin homeostasis and normal timing of lateral organ initiation at the shoot meristem in *Arabidopsis*. Plant Cell 22, 335–348. 10.1105/tpc.109.071498 20139162 PMC2845421

[B40] Loreti E. Valeri M. C. Novi G. Perata P. (2017). Gene regulation and survival under hypoxia requires starch availability and metabolism. Plant Physiol. 176, 1286–1298. 10.1104/pp.17.01002 29084901 PMC5813553

[B41] Loreti E. van Veen H. Perata P. (2016). Plant responses to flooding stress. Curr. Opin. Plant Biol. 33, 64–71. 10.1016/j.pbi.2016.06.005 27322538

[B42] Macaulay K. M. Heath G. A. Ciulli A. Murphy A. M. Abell C. Carr J. P.. (2017). The biochemical properties of the two *Arabidopsis thaliana* isochorismate synthases. Biochem. J. 474, 1579–1590. 10.1042/BCJ20161069 28356402 PMC5408348

[B43] Mamidi S. Chikara S. Goos R. J. Hyten D. L. Annam D. Moghaddam S. M. (2011). Genome-Wide association analysis identifies candidate genes associated with iron deficiency chlorosis in soybean. Plant Genome J. 4:154 10.3835/plantgenome2011.04.0011

[B44] McClean P. E. Terpstra J. McConnell M. White C. Lee R. Mamidi S. (2012). Population structure and genetic differentiation among the USDA common bean (*Phaseolus vulgaris* L.) core collection. Genet. Resour. Crop Evol. 59, 499–515. 10.1007/s10722-011-9699-0

[B45] McKenna A. Hanna M. Banks E. Sivachenko A. Cibulskis K. Kernytsky A.. (2010). The Genome Analysis Toolkit, a MapReduce framework for analyzing next-generation DNA sequencing data. Genome Res. 20, 1297–1303. 10.1101/gr.107524.110 20644199 PMC2928508

[B46] Messina M. J. (1999). Legumes and soybeans: overview of their nutritional profiles and health effects. Am. J. Clin. Nutr. 70, 439S−450S. 10.1093/ajcn/70.3.439s 10479216

[B47] Michelmore R. W. Paran I. Kesseli R. V. (1991). Identification of markers linked to disease-resistance genes by bulked segregant analysis: a rapid method to detect markers in specific genomic regions by using segregating populations. Proc. Natl. Acad. Sci. U.S.A. 88, 9828–9832. 10.1073/pnas.88.21.9828 1682921 PMC52814

[B48] Miller D. A. White R. A. (1998). A conterminous United States Multi-Layer Soil Characteristics Data Set for Regional Climate and Hydrology Modeling. Earth Interact. Available online at: http://earthinteractions.org.

[B49] Moghaddam S. M. Mamidi S. Osorno J. M. Lee R. Brick M. A. Kelly J. D. (2016). Genome-wide association study identifies candidate loci underlying agronomic traits in a middle american diversity panel of common bean (*Phaseolus vulgaris* L.). Plant Genome J. 9, 1–21. 10.3835/plantgenome2016.02.0012 27902795

[B50] Nguyen V. T. Vuong T. D. VanToai T. Lee J. D. Wu X. M. Nguyen H. T. (2012). Mapping of quantitative trait loci associated with resistance to and flooding tolerance in soybean. Crop Sci. 52:2481 10.2135/cropsci2011.09.0466

[B51] Osman K. A. Tang B. Wang Y. Chen J. Yu F. Li L.. (2013). Dynamic QTL analysis and candidate gene mapping for waterlogging tolerance at maize seedling stage. PLoS ONE 8:e79305. 10.1371/journal.pone.0079305 24244474 PMC3828346

[B52] Ou Y. Lu X. Zi Q. Xun Q. Zhang J. Wu Y.. (2016). RGF1 INSENSITIVE 1 to 5, a group of LRR receptor-like kinases, are essential for the perception of root meristem growth factor 1 in *Arabidopsis thaliana*. Cell Res. 26, 686–698. 10.1038/cr.2016.63 27229312 PMC4897188

[B53] Pritchard J. K. Stephens M. Donnelly P. (2000). Inference of population structure using multilocus genotype data. Genetics 155, 945–959. 10.1111/j.1471-8286.2007.01758.x 10835412 PMC1461096

[B54] R Development Core Team (2011). R: A Language and Environment for Statistical Computing. R Foundation Statistical Computing 1(2.11.1) (Vienna).

[B55] Ramasamy R. K. Ramasamy S. Bindroo B. B. Naik V. G. (2014). STRUCTURE PLOT: a program for drawing elegant STRUCTURE bar plots in user friendly interface. Springerplus 3:431. 10.1186/2193-1801-3-431 25152854 PMC4141070

[B56] Revelle W. (2016). Psych: Procedures for Personality and Psychological Research (Evanston, IL).

[B57] Rezaei M. Arzani A. Saeidi G. Karami M. (2017). Physiology of salinity tolerance in *Bromus danthoniae* genotypes originated from saline and non-saline areas of West Iran. Crop Pasture Sci. 68:92 10.1071/CP16311

[B58] Rojas J. A. Jacobs J. L. Napieralski S. Karaj B. Bradley C. A. Chase T.. (2017). Oomycete species associated with soybean seedlings in North America—Part II: diversity and ecology in relation to environmental and edaphic factors. Phytopathology 107, 293–304. 10.1094/PHYTO-04-16-0176-R 27841963

[B59] Rossman D. R. Rojas A. Jacobs J. L. Mukankusi C. Kelly J. D. Chilvers M. I. (2017). Pathogenicity and virulence of soilborne oomycetes on *Phaseolus vulgaris*. Plant Dis. 101, 1851–1859. 10.1094/PDIS-02-17-0178-RE 30677317

[B60] Sayama T. Nakazaki T. Ishikawa G. Yagasaki K. Yamada N. Hirota N.. (2009). QTL analysis of seed-flooding tolerance in soybean (*Glycine max* [L.] Merr.). Plant Sci. 176, 514–521. 10.1016/j.plantsci.2009.01.007 26493141

[B61] Schröder S. Mamidi S. Lee R. McKain M. R. McClean P. E. Osorno J. M. (2016). Optimization of genotyping by sequencing (GBS) data in common bean (*Phaseolus vulgaris* L.). Mol. Breed. 36, 1–9. 10.1007/s11032-015-0431-1

[B62] Setter T. L. Laureles E. V. (1996). The beneficial effect of reduced elongation growth on submergence tolerance of rice. J. Exp. Bot. 47, 1551–1559. 10.1093/jxb/47.10.1551

[B63] Singh S. P. Gepts P. Debouck D. G. (1991). Races of common bean (*Phaseolus vulgaris*, Fabaceae). Econ. Bot. 45, 379–396. 10.1007/BF02887079

[B64] Singh S. P. Terán H. Lema M. Webster D. M. Strausbaugh C. A. Miklas P. N. (2007). Seventy-five years of breeding dry bean of the western USA. Crop Sci. 47, 981–989. 10.2135/cropsci2006.05.0322

[B65] Soltani A. MafiMoghaddam S. Walter K. Restrepo-Montoya D. Mamidi S. Schroder S.. (2017). Genetic architecture of flooding tolerance in dry bean Middle-American diversity panel. Front. Plant Sci. 8:1183. 10.3389/fpls.2017.01183 28729876 PMC5498472

[B66] Soma F. Mogami J. Yoshida T. Abekura M. Takahashi F. Kidokoro S.. (2017). ABA-unresponsive SnRK2 protein kinases regulate mRNA decay under osmotic stress in plants. Nat. Plants 3:16204. 10.1038/nplants.2016.204 28059081

[B67] Steffens B. Rasmussen A. (2016). The physiology of adventitious roots. Plant Physiol. 170, 603–617. 10.1104/pp.15.01360 26697895 PMC4734560

[B68] Sun G. Zhu C. Kramer M. H. Yang S. S. Song W. Piepho H. P.. (2010). Variation explained in mixed-model association mapping. Heredity 105, 333–340. 10.1038/hdy.2010.11 20145669

[B69] Tournaire-Roux C. Sutka M. Javot H. Gout E. Gerbeau P. Luu D. T.. (2003). Cytosolic pH regulates root water transport during anoxic stress through gating of aquaporins. Nature 425, 393–397. 10.1038/nature,01853 14508488

[B70] USDA-NRCS. (2017). Web Soil Survey. USDA-NRCS. Available online at: https://websoilsurvey.sc.egov.usda.gov/App/HomePage.htm

[B71] Valliyodan B. Ye H. Song L. Murphy M. Shannon J. G. Nguyen H. T. (2016). Genetic diversity and genomic strategies for improving drought and waterlogging tolerance in soybeans. J. Exp. Bot. 68, 1835–1849. 10.1093/jxb/erw433 27927997

[B72] VanToai T. T. St Martin, S. K. Chase K. Boru G. Schnipke V. Schmitthenner A. F. (2001). Identification of a QTL associated with tolerance of soybean to soil waterlogging. Crop Sci. 41, 1247–1252. 10.2135/cropsci2001.4141247x

[B73] Visscher P. M. Hill W. G. Wray N. R. (2008). Heritability in the genomics era — concepts and misconceptions. Nat. Rev. Genet. 9, 255–266. 10.1038/nrg2322 18319743

[B74] Wei T. Simko V. (2017). R package corrplot: Visualization of a Correlation *Matrix*. Available online at: https://github.com/taiyun/corrplot

[B75] White T. Bruns T. Lee S. Taylor J. (1990). Amplification and direct sequencing of fungal ribosomal RNA genes for phylogenetics, in PCR Protocols: A Guide to Methods and Applications, eds Innis M. Gelfand D. Shinsky J. White T. (San Diego, CA: Academic Press, inc.), 315–322.

[B76] Xu J. Wang X. Guo, W-Z. (2015). The cytochrome P450 superfamily: Key players in plant development and defense. J. Integr. Agric. 14, 1673–1686. 10.1016/S2095-3119(14)60980-1

[B77] Yang H. Liu J. Lin J. Deng L. Fan S. Guo Y.. (2016). Overexpression of CHMP7 from rapeseed and *Arabidopsis* causes dwarfism and premature senescence in *Arabidopsis*. J. Plant Physiol. 204, 16–26. 10.1016/j.jplph.2016.06.023 27497741

[B78] Zhao J. H. (2007). Gap : genetic analysis package. J. Stat. Softw. 23, 1–18. 10.18637/jss.v023.i08

[B79] Zhao Y. Christensen S. K. Fankhauser C. Cashman J. R. Cohen J. D. Weigel D.. (2001). A role for flavin monooxygenase-like enzymes in auxin biosynthesis. Science 291, 306–309. 10.1126/science.291.5502.306 11209081

