# Supplementary figures and images for: Genetic Analysis of Flooding Tolerance in an Andean Diversity Panel of Dry Bean (Phaseolus vulgaris L.)
Source: Front Plant Sci. 2018 Jun 6;9:767. doi: 10.3389/fpls.2018.00767 (PMC5997968; doi:10.3389/fpls.2018.00767)

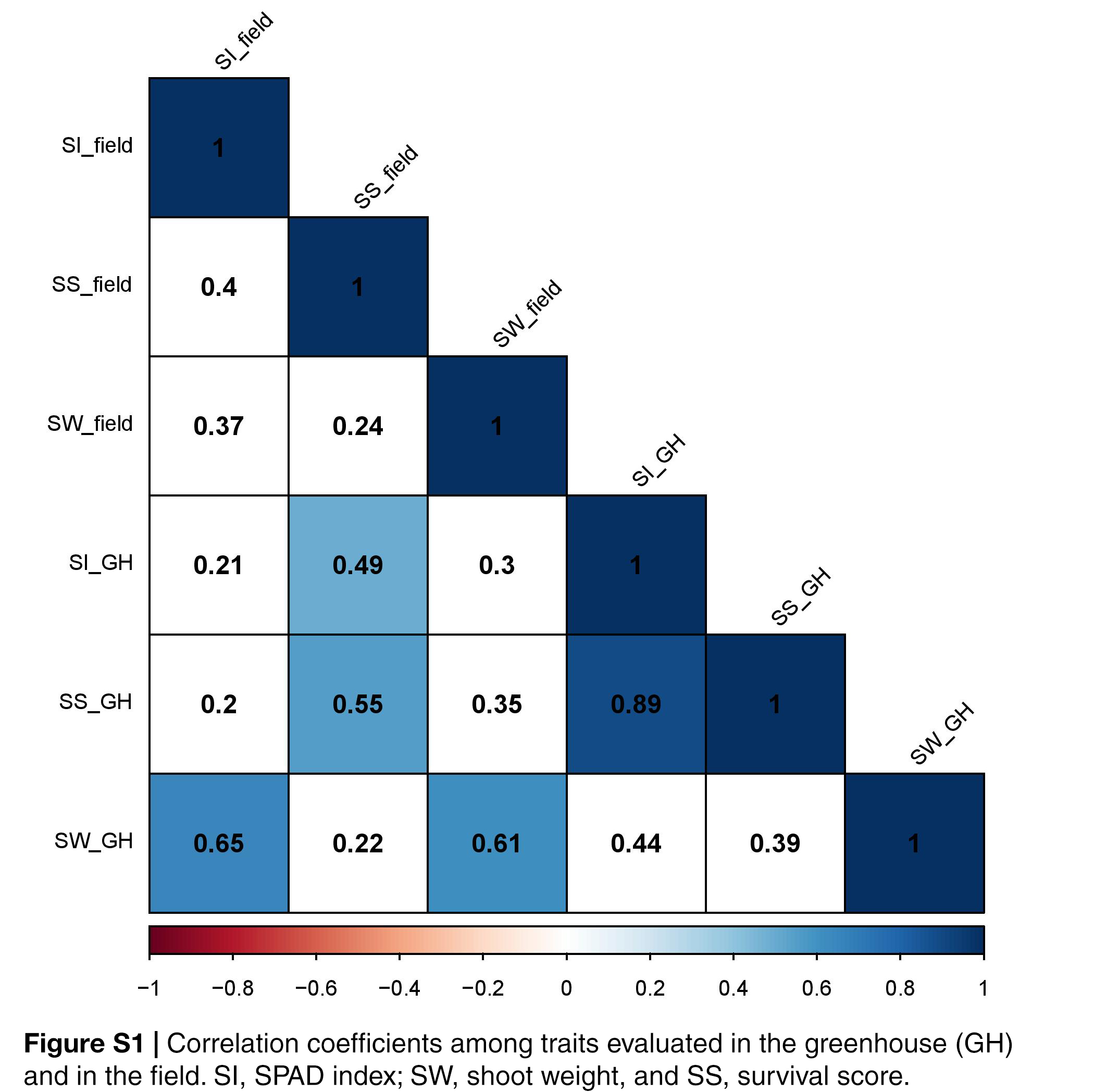

Supplement: Supplementary file 1 [file Image_1.JPEG]

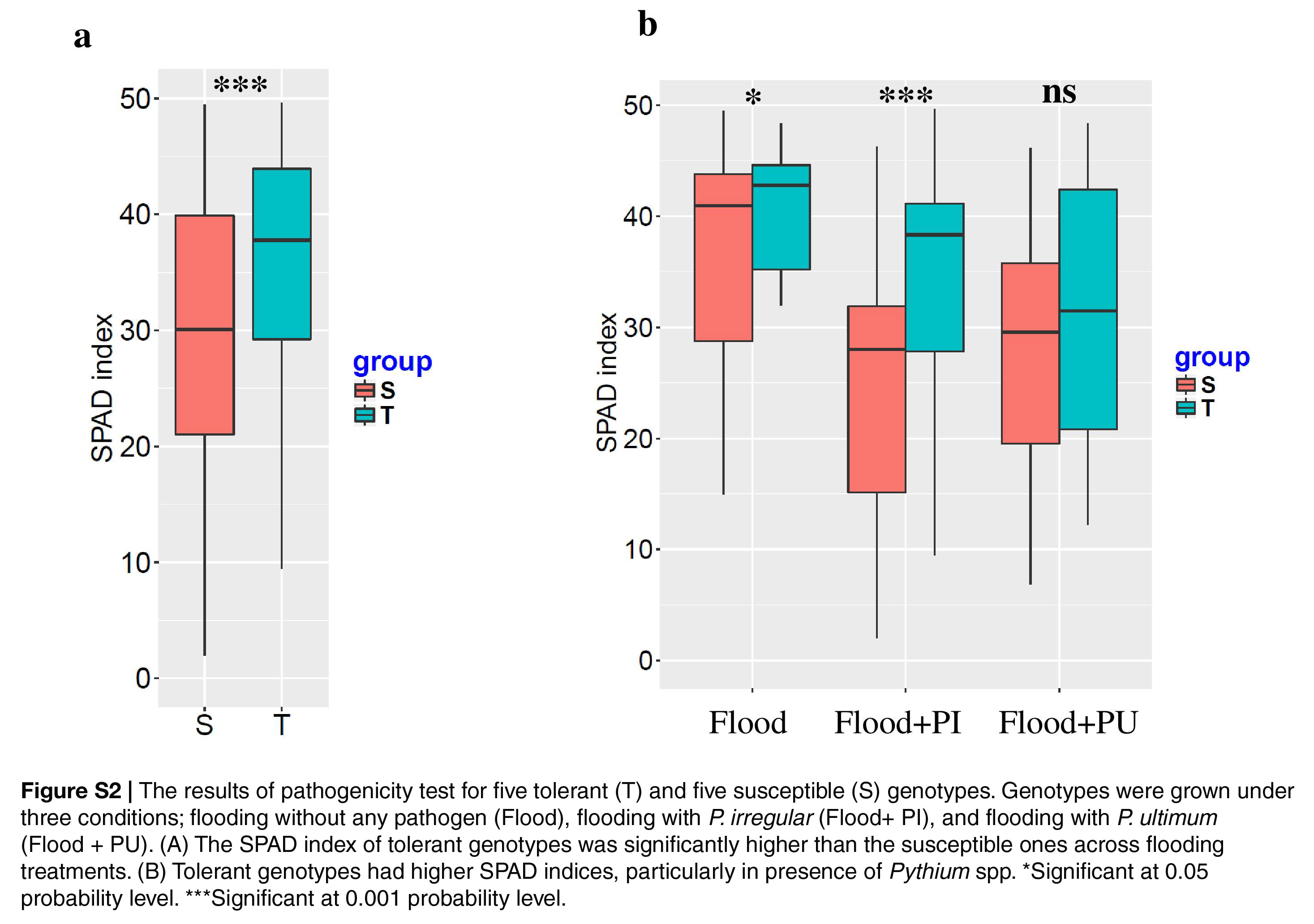

Supplement: Supplementary file 2 [file Image_2.JPEG]
