# Supplementary material for: Genetic Analysis of Flooding Tolerance in an Andean Diversity Panel of Dry Bean (Phaseolus vulgaris L.)
Source: Front Plant Sci. 2018 Jun 6;9:767. doi: 10.3389/fpls.2018.00767 (PMC5997968; doi:10.3389/fpls.2018.00767)
Supplement: Supplementary file 3 [file Table_1.docx]

| Table S1. List of the most tolerant genotypes at germination stage. | | | | | |
| --- | --- | --- | --- | --- | --- |
| Genotype ID | Genotype name | Seed type | Collection region | Germination rate  (%, well-drained) | Germination rate  (%, flooded) |
| ADP-429 | PR9920-171 | Cranberry | Caribbean | 100 | 66 |
| Check | Royalty | Cream | US core | 93 | 63 |
| ADP-610 | G-122 | Cranberry | North America | 93 | 63 |
| ADP-683 | Intermediate Jamaican red | Pink mottled | North America | 97 | 60 |
| ADP-515 | Katarina Kibala | Cranberry | Angola | 90 | 60 |
| ADP-383 | PI209486 | Red mottled | US core | 76 | 56 |
| ADP-673 | UC Nichols | Dark red kidney | North America | 97 | 53 |
| ADP-033 | KIJIVU | Purple speckled | Africa | 100 | 53 |
| ADP-650 | K42 | Light red kidney | North America | 90 | 50 |
| ADP-460 | PI331356B | Purple mottled | East Africa | 87 | 50 |
| ADP-027 | NA | Mottled | Africa | 93 | 49 |
| ADP-102 | Jesca | Purple speckled | Africa | 100 | 49 |

| Table S2. List of the most tolerant genotypes at seedling stage. Genotypes were chosen based on the selection index calculated by equation 2 | | | | |
| --- | --- | --- | --- | --- |
| Genotype ID | Genotype name | Seed type | Collection region | Selection index |
| ADP-604 | 1062 V98 | Light red kidney | North America | 13.00 |
| ADP-650 | K42 | Light red kidney | North America | 12.00 |
| ADP-673 | UC Nichols | Dark red kidney | North America | 11.72 |
| ADP-672 | CDRK | Dark red kidney | North America | 11.64 |
| ADP-687 | Pink Panther | Light red kidney | North America | 11.49 |
| ADP-686 | UCD707 | Yellow | North America | 11.40 |
| ADP-662 | USCR9 | Cranberry | North America | 11.14 |
| ADP-446 | Raz25 | Red mottled | Caribbean | 11.04 |
| Check | Royalty | Cream | US core | 10.62 |
| ADP-651 | K59 | Light red kidney | North America | 10.45 |
| ADP-431 | Gurabo5 | Cranberry | Caribbean | 10.42 |
| ADP-640 | Beluga | White kidney | North America | 10.35 |
| ADP-346 | G22246 | Red mottled | CIAT core | 10.33 |
| ADP-680 | Clouseau | Light red kidney | North America | 10.28 |
| ADP-603 | Wallace773 V98 | Light red kidney | North America | 10.15 |
| ADP-392 | PI309701 | Cranberry | US core | 10.08 |
| ADP-337 | G21303 | White kidney | CIAT core | 10.00 |

| Table S3. Number of individuals assigned to each group based on different values of K. | | | | | | | | | | |
| --- | --- | --- | --- | --- | --- | --- | --- | --- | --- | --- |
|  | | K=2 | | K=3 | | | K=4 | | | |
|  | | Middle America | Andean | Middle America | Africa | North America | Tanzania | Africa | North America | Middle America |
| No. individuals | | 20 | 257 | 20 | 190 | 67 | 18 | 173 | 67 | 20 |
| No. pure individuals † | | 19 | 249 | 19 | 145 | 53 | 14 | 111 | 44 | 19 |
| Origin % | |  |  |  |  |  |  |  |  |  |
|  | Africa | 50 | 54 | 50 | 72 | 0 | 100 | 71 | 0 | 50 |
|  | North America | 0 | 33 | 0 | 11 | 89 | 0 | 12 | 89 | 0 |
|  | Central America | 35 | 6 | 35 | 9 | 7 | 0 | 8 | 7 | 35 |
|  | South America | 5 | 4 | 5 | 5 | 1 | 0 | 6 | 1 | 5 |
|  | Eurasia | 10 | 3 | 10 | 3 | 3 | 0 | 3 | 3 | 10 |
| † individuals with Q values of more than 0.8 were considered as “pure”. | | | | | | | | | | |

| Table S4. Effect of flooding stress on a subset of Andean beans under the field conditions in 2016. | | | | | | | |
| --- | --- | --- | --- | --- | --- | --- | --- |
|  | Non-flooded | | |  | Flooded | | |
|  | Mean ± SE |  | Range |  | Mean ± SE |  | Range |
| Survival score | 4.53 ± 0.08 |  | 3.67-5.00 |  | 1.74 ± 0.16 |  | 0.83-3.50 |
| SPAD index | 44.90 ± 0.96 |  | 37.57-51.43 |  | 26.13 ± 1.27 |  | 12.67-36.43 |
| Shoot weight | 2.75 ± 0.13 |  | 1.89-4.48 |  | 0.13 ± 0.05 |  | 0.35-1.37 |

| Table S5. Pythium isolates from beans grown in two different soils under flooding condition. Isolates were identified with BLASTn. | | | | | |
| --- | --- | --- | --- | --- | --- |
| Isolate | Closest Match | % Identity | NCBI Accession | Soil Source | Number of cultures |
| Pyth.1 | *Pythium irregulare* | 98 | [AY907910.1](https://www.ncbi.nlm.nih.gov/nucleotide/60100323?report=genbank&log$=nucltop&blast_rank=1&RID=R4STSXE5014) | Michigan | 8 |
| Pyth.2 | *Pythium irregulare* | 96 | [AY907910.1](https://www.ncbi.nlm.nih.gov/nucleotide/60100323?report=genbank&log$=nucltop&blast_rank=1&RID=R4STSXE5014) | Michigan | 4 |
| Pyth.3 | *Pythium ultimum* | 95 | [LT670911.1](https://www.ncbi.nlm.nih.gov/nucleotide/1111510529?report=genbank&log$=nucltop&blast_rank=1&RID=0KXY66AC014) | North Dakota | 5 |
| Pyth.4 | *Pythium sylvaticum* | 97 | [KU211487.1](https://www.ncbi.nlm.nih.gov/nucleotide/1063194030?report=genbank&log$=nucltop&blast_rank=1&RID=0KXY66AC014) | North Dakota | 1 |

| Table S6. List of potential candidate genes, associated with major significant loci, detected in flooded stress | | | | | | | |
| --- | --- | --- | --- | --- | --- | --- | --- |
| Trait† | Locus | Distance | *Phaseolus* gene | Best *Arabidopsis* hit | Gene ID | Function | Reference |
| GR | Pv06/16.0 | -2,047 | Phvul.006G054700 | AT3G26300.1 | CYP71B34 | Cytochrome P450, involved in oxidation-reduction process | XU et al., 2015 |
|  | Pv08/3.2 | -1,401 | Phvul.008G039400 | AT3G01090.2 | KIN10,SnRK1.1 | Involved in regulatory response under hypoxic condition | Baena-González et al., 2007 |
| TW | Pv05/1.2 | 3,456 | Phvul.005G013500 | AT2G34660.1 | MRP2 | Involved in cytosolic detoxification | Frelet-Barrand et al., 2008 |
|  | Pv08/59.1 | 25,677 | Phvul.008G241900 | AT5G56550.1 | OXS3 | Involved in tolerance to heavy metal and oxidative stress | Blanvillain et al., 2009 |
| SW | Pv05/1.2 | 3,456 | Phvul.005G013500 | AT2G34660.1 | MRP2 | Involved in cytosolic detoxification | Frelet-Barrand et al., 2008 |
|  | Pv08/59.1 | 25,677 | Phvul.008G241900 | AT5G56550.1 | OXS3 | Involved in tolerance to heavy metal and oxidative stress | Blanvillain et al., 2009 |
| RW | Pv08/62.3 | 43,391 | Phvul.008G281500 | AT5G56040.2 | RGI4 | Root meristem development | Ou et al., 2016 |
|  | Pv09/13.5 | 9,680 | Phvul.009G081900 | AT4G33210.1 | SLOMO | Auxin Homeostasis, lateral root formation | Lohmann et al., 2010 |
|  |  | 31,656 | Phvul.009G081800 | AT5G21482.1 | ATCKX5,CKX7 | Cytokinin catalyze, root development | Köllmer et al., 2014 |
| HL | Pv07/24.4 | 79,637 | Phvul.007G147400 | AT2G36910.1 | ABCB1,ATPGP1,PGP1 | Establishing proper auxin gradient and hypocotyl elongation | Ge et al., 2017 |
|  | Pv09/7.8 | 72,124 | Phvul.009G036000 | AT4G24490.2 | RGTA1 | Hypocotyl length | Hála et al., 2010 |
| SI | Pv06/24.3 | -12,572 | Phvul.006G135300 | AT3G13300.2 | VARICOSE | Osmotic stress response | Soma et al., 2017 |
|  | Pv08/61.1 | -34,361 | Phvul.008G265400 | AT3G47860.1 | CHL | Chloroplastic lipocalin, thylakoidal membrane protection | Levesque-Tremblay et al., 2009 |
| AR | Pv07/28.7 | 28,267 | Phvul.007G170100 | AT3G63110.1 | IPT3 | Cytokinin biosynthesis, lateral root formation | Chang et al., 2013 |
|  | Pv08/62.3 | 43,391 | Phvul.008G281500 | AT5G56040.2 | RGI4 | Root meristem development | Ou et al., 2016 |
|  | Pv09/13.5 | 9,680 | Phvul.009G081900 | AT4G33210.1 | SLOMO | Auxin Homeostasis, lateral root formation | Lohmann et al., 2010 |
|  |  | 31,656 | Phvul.009G081800 | AT5G21482.1 | ATCKX5,CKX7 | Cytokinin catalyze, root development | Köllmer et al., 2014 |
|  | Pv09/20.2 | 70,095 | Phvul.009G133300 | AT1G21430.1 | YUC11 | Auxin biosynthesis, root formation | Zhao et al., 2001 |
| SC | Pv02/47.3 | -31,308 | Phvul.002G305900 | AT2G19830.1 | SNF7.2 | Involved in leaf senescence in interaction with CHMP7 | Yang et al., 2016 |
|  | Pv04/46.8 | 30,151 | Phvul.004G163900 | AT4G13250.1 | NYC1 | Involved in chlorophyll b degradation and leaf senescence | Horie et al., 2009 |
|  | Pv09/19.1 | -74,794 | Phvul.009G125900 | AT1G01720.1 | ATAF1 | Transcription factor controlling leaf senescence | Garapati et al., 2015 |
|  | Pv10/1.5 | -72,600 | Phvul.010G011700 | AT1G18870.1 | ICS2 | Salicylic acid biosynthesis and defense mechanism | Macaulay et al., 2017 |
| † Abbreviate name of the trait. GR: germination rate, TW: total weight, SW: shoot weight, RW: root weight, HL: hypochotyl length, SI: SPAD index, AR: adventitious rate and SR: survival score | | | | | | | |
